# Supplementary material for: A High Separation Factor for 165Er from Ho for Targeted Radionuclide Therapy
Source: Molecules. 2021 Dec 11;26(24):7513. doi: 10.3390/molecules26247513 (PMC8707915; doi:10.3390/molecules26247513)
Supplement: Supplementary file 1 [file molecules-26-07513-s001.zip › molecules-1477506-supplementary.pdf]

Supporting Information:
A high separation factor for ^{165}Er from Ho for targeted radionuclide therapy

I. Da Silva[a,b], T. R. Johnson[a], J. C. Mixdorf[a], E. Aluicio-Sarduy[a], T. E. Barnhart[a], R. J. Nickles[a], J. W. Engle[a,c], P. A. Ellison[a]

[a]Department of Medical Physics, University of Wisconsin School of Medicine and Public Health 1111 Highland Avenue Madison, WI 53705
[b,]CEMHTI, CNRS, UPR3079, Univ. Orléans, F-45071 Orléans France
[c]Department of Radiology, University of Wisconsin School of Medicine and Public Health 1111 Highland Avenue Madison, WI 53705

Table of Contents

SUPPLEMENTARY FIGURE S1. 2
SUPPLEMENTARY FIGURE S2. 3
SUPPLEMENTARY MATERIAL SECTION 1 4
SUPPLEMENTAL FIGURE S3 5
SUPPLEMENTARY MATERIAL SECTION 2 6
SUPPLEMENTARY FIGURE S4 7
SUPPLEMENTARY MATERIAL SECTION 3 8
SUPPLEMENTARY FIGURE S5 9
SUPPLEMENTARY FIGURE S7 12
SUPPLEMENTARY FIGURE S8 13
SUPPLEMENTARY EQUATION S1 14
SUPPLEMENTAL MATERIAL REFERENCES 15

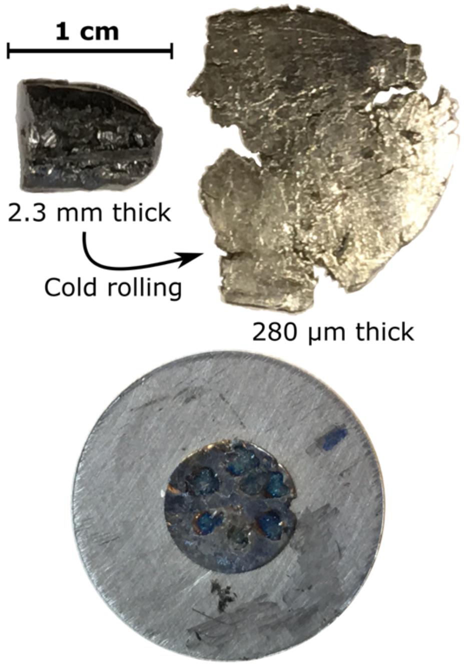

Supplementary Figure S1. Top: 9.1 by 7.3 by 2.3 mm thick Ho piece before and after cold rolling to 16.5 by 19.7 by 0.28 mm thick foil with visible cracking around edges. Bottom: Holmium (122.2 mg, 7.9 mm diameter, 280 µm thick, 0.5 ppm Er impurity) spot welded to 19 mm diameter, 500 µm thick Ta.

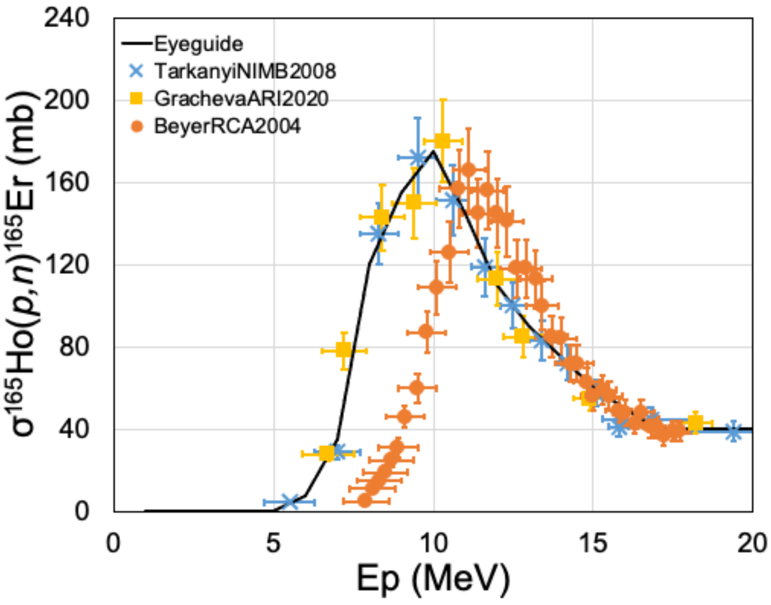

Supplementary Figure S2. ^{165}Ho(*p,n*)^{165}Er excitation function with experimental data from TarkanyiNMB2008 [1] (blue ╳), GrachevaARI2020 [2] (yellow square), BeyerRCA2004 [3] (orange circle). Cross sections shown in the black line eyeguide were used for theoretical ^{165}Er yield calculations.

Supplementary material section 1: Investigation of loading capacity of LN2 EXC columns

Purpose

To determine the impact of holmium loading mass on LN2 extraction chromatography columns.

Materials and Methods

A holmium target (174 mg, outer diameter 6.35mm, thickness 690µm, Alfa Aesar, 99.9%, 0.06% Er content) was irradiated with a CTI RDS112 cyclotron at 11 MeV proton for 2 h with an average proton current of 14 µA. It was dissolved in 11 M HCl (2 mL) and dried with heat and argon flow. After cooling, the pink salts were dissolved in 0.07 M alpha-hydroxyisobutyric acid (4 mL, pH = 4.7, αHIBA, Sigma Aldrich). This was the Ho/^{165}Er stock solution. 16 M HNO_3 (TraceSelect, Fluka) was used to acidify solution or to prepare diluted nitric acid solutions (0.4M and 1M) with 18 MΩ·cm ultrapure water (Milli-Q). LN2 resin from Triskem (20-50 µm) has a capacity of 0.16 mmol/ml of resin and density of 0.37 g/ml. A 5.5 mm inner diameter polypropylene column filled with LN2 resin (300 mg) was used for all experiments. Before each attempt, LN2 resin had been preconditioned with 5 M HNO_3 (5 mL) then water (40 mL) (checking neutral pH value) followed by 0.1 M HNO_3 (5 mL). Separations were performed by loading 1 mg (5% theoretical maximum capacity), 3 mg (14% capacity), and 10 mg (47% capacity) holmium and 0.6 – 1.5 MBq ^{165}Er diluted in 0.1 M HNO_3 (200 mL), 0.07 M αHIBA to simulate recovered ^{165}Er-rich fraction from the CX/αHIBA separation. The column was loaded at 7.3 ± 0.4 ml/min (n = 3), followed with 28.6 ± 0.8 ml (n = 3) 0.4 M HNO_3 at 1.0 ± 0.2 ml/min (n = 3) to elute holmium before switching the eluent to 1 M HNO_3 to elute rest of ^{165}Er on resin.

For calculations of Ho/Er separation factor ($SF_{Ho/Er}$), ^{165}Er was quantified by a radioactivity dose calibrator (Capintec CRC-15R, setting #260) and Holmium by MP-AES (Agilent MP4200). MP-AES was done on samples diluted 10x in 0.1 M HCl. Under these conditions, MP-AES limit of detection (LD) is 1 ppm. All measurements above below value were estimated as less than or equal to 1 ppm for $SF_{Ho/Er}$. To assess whether the increased loading mass affected chromatographic performance under the optimized conditions presented in the main manuscript, the $SF_{Ho/Er}$ was measured based on the assumption that the column would be rinsed with 0.4 M HNO_3 until 20% of the ^{165}Er had eluted, followed by the recovery of the remaining ^{165}Er for DGA EXC work up. For each of these three experiments, the $SF_{Ho/Er}$ was calculated according to equation (1) based on the assumption that all fractions recovered after the elution of the first 20% of ^{165}Er were pooled into a single fraction, resulting in ~80% ^{165}Er recovery.

Results and Discussion

The elution profiles from the three LN2 EXC columns are shown in **Figure S3**. At the lowest loaded holmium mass (1 mg, 5% theoretical max capacity, **Figure S3** top), the Ho/^{165}Er elution profiles appear similar to those performed under optimized conditions (see **Figures 3** and **S4** as examples). When recovering 80% ^{165}Er by collecting all fractions after the vertical red arrow, the separation resulted in a $SF_{Ho/Er}$ = 140. Upon increasing the loaded Ho mass (3 mg, 14% max capacity, **Figure S3** middle), the holmium and ^{165}Er were observed to elute significantly earlier. This resulted in a decrease in column $SF_{Ho/Er}$ = 11. This trend continued when the column was loaded with 10 mg Ho (47% max capacity, **Figure S3** bottom) with the holmium and ^{165}Er eluting even earlier and a further decreased column $SF_{Ho/Er}$ = 4.5. It is hypothesized that increasing Ho

loading mass limits the number of free sites for extraction on the resin. This results in a significant breakthrough of both holmium and erbium and decrease in chromatographic performance.

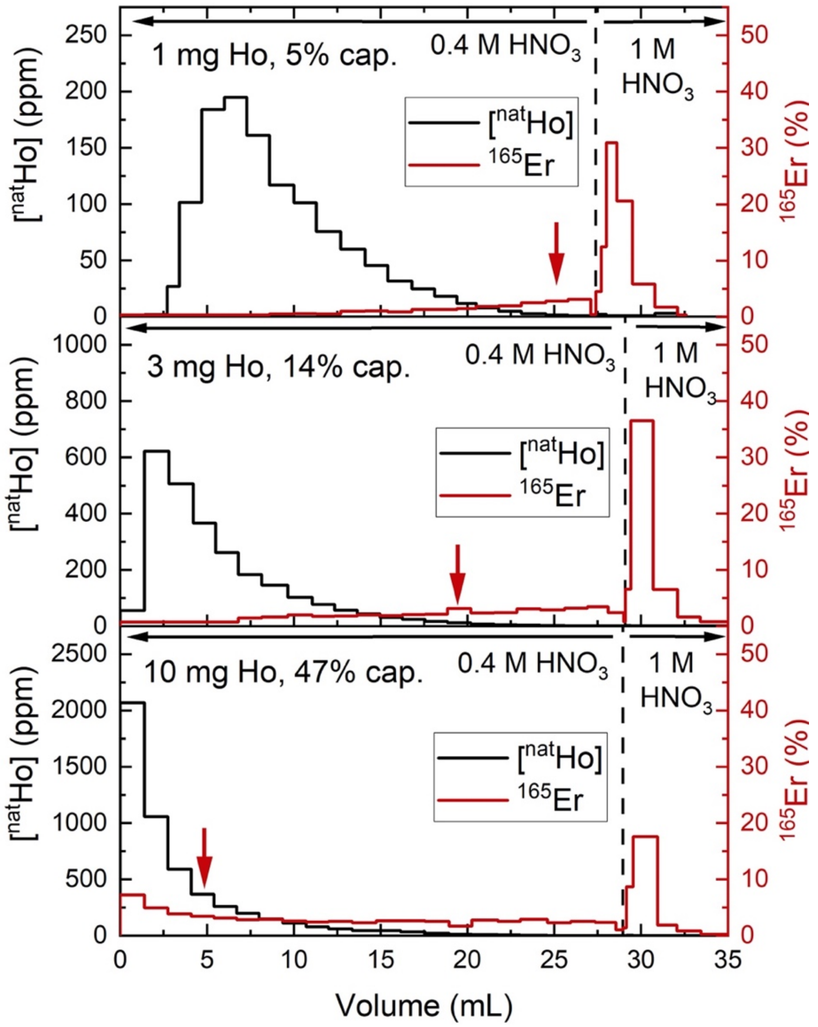

Supplemental Figure S3. Holmium (black lines, and ^{165}Er (red lines, elution profiles from 300 mg LN2 columns loaded with 1 mg (top), 3 mg (middle) or 10 mg (bottom) holmium, 0.6 – 1.5 MBq ^{165}Er in 200 mL 0.1 M HNO3, 70 mM αHIB and eluted with ~29 mL 0.4 M HNO_3, followed by ~5 mL 1 M HNO_3.

__Conclusion__

These experiments demonstrate that under the investigated conditions the chromatographic performance of LN2 EXC columns is significantly decreased when holmium loading masses are increased from 5% theoretical maximum capacity to 14% to 47%. This conservatively limits the loading capacity of the 500 mg LN2 columns used in the bulk of this work to 1 – 2 mg (3 – 6% theoretical max capacity) of holmium.

Supplementary material section 2. Effect of dry loading conditions on Ho/Er chromatographic performance on LN2 EXC resin columns.

Purpose

Because of the high flow rate of the loading process, there have been incidences of the LN2 column going dry between the loading and rinsing steps. As column dryness could create channelling within the column resin that allows eluents to pass through with decreased interaction, this experiment compared two identical LN2 columns, one run dry and the other not, to determine if dryness during the loading step hindered Ho-Er separation.

Materials and methods

This testing of the LN2 column required the intermediate step of the full Ho-^{165}Er separation procedure. The columns were freshly dry packed using 500 mg of LN2 (20-50 μm, Triskem Int.) in a 1mL fritted polypropylene column. All columns were preconditioned with 1 M HNO_3 (5 mL) then 0.1 M HNO_3 (25 mL) with all nitric acid solutions made with 16 M HNO_3 (TraceSelect, Fluka) and 18 MΩ·cm ultrapure water (Milli-Q). Holmium was taken from a solution with a known concentration of 100 mg Ho in 0.07 M α-HIBA (44.4 mL), and the erbium from 10 mg of $ErCl_3$ dissolved in 10 mL of Milli-Q water. A loading solution of 0.07 M α-HIBA (220mL, pH 2.6), 0.5 mg Ho (220uL), and 0.5 mg $ErCl_3$ (500uL) was used for each trial. A peristaltic pump (Welco Co. Ltd, WPM1-P1CA-WP) loaded the solution at a rate of 6 mL/min onto the LN2 column. Under one experimental condition, deemed 'wet', the column was loaded but still had liquid in the lines when switched to the rinsing phase. Under a second experimental condition, deemed 'dry', the column was loaded, and the pump ran until the loading solution had drained from the column and loading lines. Once loaded, another peristaltic pump (Welco Co. Ltd, Welco Co. Ltd, WPM1-P1BB-BP) rinsed the columns with 0.4 M HNO_3 (100 mL) at 1 mL/min collected in 5 mL fractions, followed by 5 M HNO_3 (4 mL). The conditioning, loading, and rinsing were repeated to obtain two identical replicates for each experimental condition.

The amount of holmium and erbium in each fraction was quantified using MP-AES (Agilent MP4200). Holmium standard solutions of 0.05 – 50 ppm were made by dissolving holmium metal in 11 M HCl, followed by dilution in 0.1 M HCl. Similarly, erbium standards were created by dissolving erbium chloride in 0.1 M HCl and diluted to the necessary concentrations. From each fraction (one loading, twenty rinsing, and one elution), 100 μL was taken and diluted to 1 mL with 0.1M HCl. The limit of detection for holmium and erbium was estimated to be ~1 ppm in these diluted sample solutions.

Results and Discussion

The MP-AES data showed holmium and erbium following the expected elution pattern, though some fractions had below detection limit concentrations (**Figure S4**). Average and standard deviation error bars are reported for the duplicated experiments under each experimental condition. Even with values being recorded as the limit of detection, the columns run dry appear to have a broader holmium elution peak along with erbium eluting sooner, therefore more peak overlap. As

the goal of the LN2 column is to recover as much erbium and as little holmium as possible, the less these peaks overlap, the higher the erbium recovery while maintaining an adequate Ho/Er separation factor. Though the MP-AES detection limit obfuscates any quantifiable difference between the two experimental conditions in terms of Ho/Er separation factor, the peak overlap with non-overlapping error bars supports the idea that running the column dry may lead to a less successful separation.

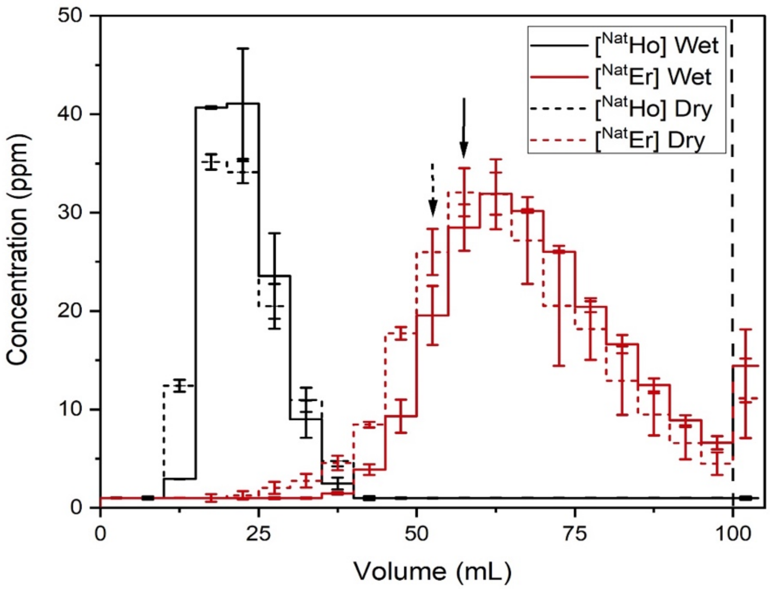

Supplementary figure S4. Elution profiles (n = 2 for each run type) for holmium (black) and erbium (red) eluted in rinse and elution fractions from 500 mg resin LN2 columns. The solid lines refer to the "wet run" trials where the column and lines were kept wet between the loading and rinsing stage. The dashed vertical line indicates the change between the rinsing (0.4 M HNO_3) and elution (1 M HNO_3) stage. The vertical black arrows indicate the fraction in which the threshold of 20% erbium collection was reached. The error bars show the standard deviation between the two trials.

Conclusions

When using a freshly packed LN2 column, running the column dry may impact chromatographic performance. However, because the MP-AES quantified fraction concentrations in this experiment were near or below the limit of detection, the effect, if present, is only mild and does not render the separation useless if column dryness occurs.

Supplementary material section 3. Effect of column reuse and dry loading conditions on Ho/Er chromatographic performance on LN2 EXC resin columns.

Purpose

To expand on the impact of dryness on the LN2 column, an experiment was run that explored if long durations of dry storage would cause changes in the resin thereby not allowing for reuse. This experiment took three columns stored dry from use in previous separations, ran them dry during the loading stage, and examined erbium and holmium concentrations of rinse fractions to determine if high quality separation was achievable.

Materials and methods

This testing of the LN2 column required the intermediate step of the full Ho-^{165}Er separation procedure. The columns containing LN2 resin (500 mg, 20-50 µm, Triskem Int.) in a 1mL fritted polypropylene column were used at least once in previous ^{165}Er radioisolation chemistry, then stored dry for 20, 11, or 3 weeks. All three columns were then reconditioned with 1 M HNO_3 (5 mL) then 0.1 M HNO_3 (25 mL) with all nitric acid solutions made with 16 M HNO_3 (TraceSelect, Fluka) and 18 MΩ·cm ultrapure water (Milli-Q). Holmium was taken from a solution with a known concentration of Ho (100 mg) in 0.07 M α-HIBA (44.4 mL), and the erbium from $ErCl_3$ (10 mg) dissolved in Milli-Q water (10 mL). A loading solution of 0.07 M α-HIBA (220 mL, pH 2.6), Ho (0.5 mg, 220 µL), and $ErCl_3$ (0.5 mg, 500 µL) was used for each trial. A peristaltic pump (Welco Co. Ltd, WPM1-P1CA-WP) loaded the solution at a rate of 6 mL/min onto the LN2 column until the solution was fully loaded and had drained from the column and loading lines. Once loaded, another peristaltic pump (Welco Co. Ltd, WPM1-P1BB-BP) rinsed the column with 0.4 M HNO_3 (100 mL) at 1 mL/min collected in 5 mL fractions followed by 5 M HNO_3 (4 mL).

The amount of holmium and erbium in each fraction was quantified using MP-AES (Agilent MP4200). Holmium standard solutions of 0.05 – 50 ppm were made by dissolving holmium metal in 11 M HCl, followed by dilution in 0.1 M HCl. Similarly, erbium standards were created by dissolving erbium chloride in 0.1 M HCl and serially diluted to the necessary concentrations. From each fraction (one loading, twenty rinsing, and one elution), 100 µL was taken and diluted to 1 mL with 0.1M HCl. The limit of detection for holmium and erbium was estimated to be ~1 ppm in these diluted sample solutions.

Results and Discussion

Elution profiles for the three run-dry reused columns are shown in **Figure S5**. There was a marked difference in the elution patterns from both the standard LN2 performance (**Figures 3, S4**) and from each other. Instead of a gradual peak of holmium elution followed by a peak of erbium elution, the elements washed out in unpredictable patterns, the erbium barely eluting until the elution fraction for two of the columns. Though this experiment did not repeat trials for each column, the varied elution patterns between dry columns preconditioned and run the same way made the results unpredictable and likely not repeatable. This evidence supports the idea that

reusing columns stored dry and running them dry in the loading process causes significant separation issues.

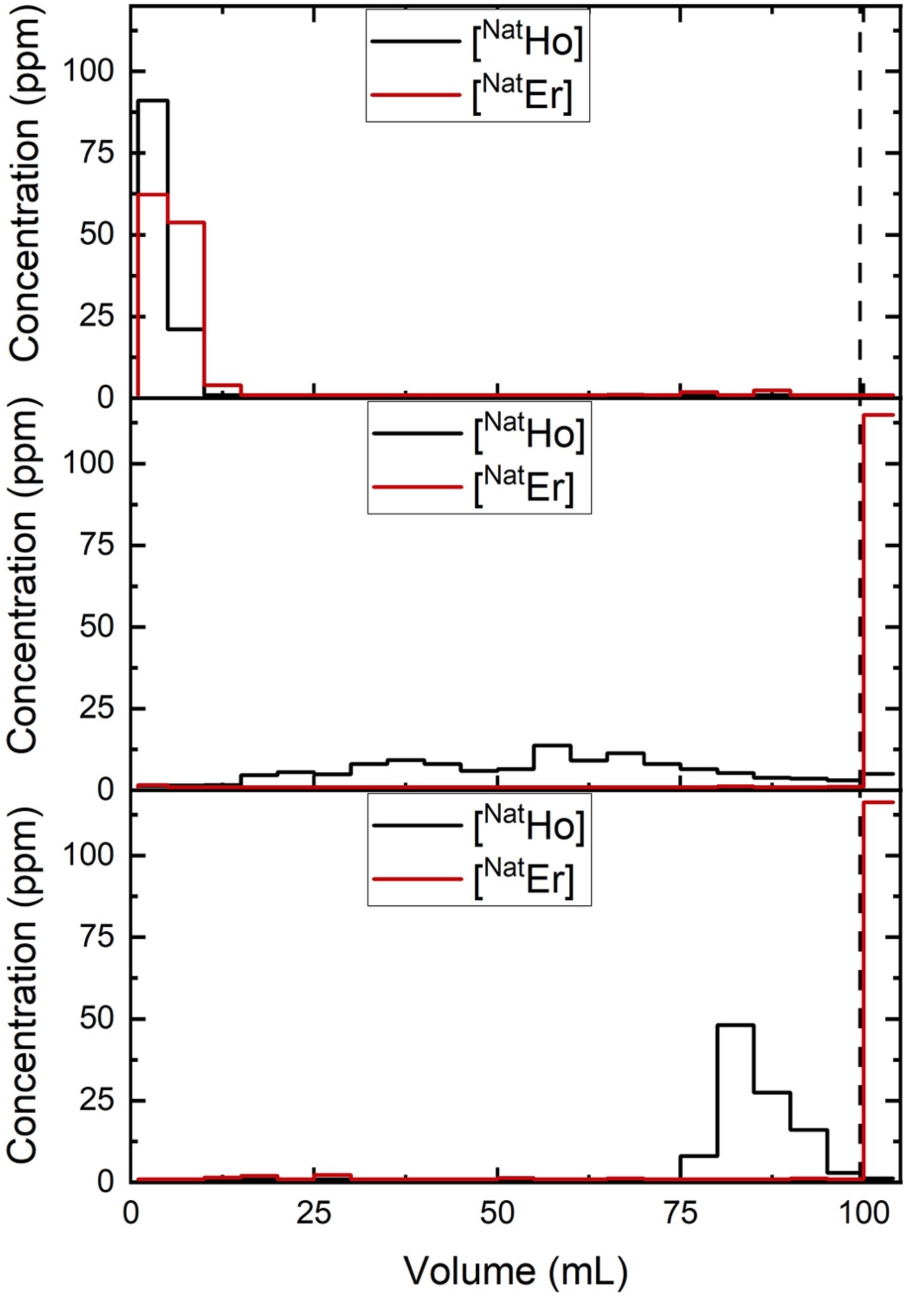

Supplementary Figure S5. Elution profile for holmium (black) and erbium (red) eluted in rinse and elution fraction for reused 500 mg LN2 columns stored dry 3 (top), 11 (middle), and 20 (bottom) weeks. The concentrations of Ho and Er for many of the fractions were near the detection limit. The dashed vertical line indicates the change between the rinsing (0.4 M HNO_3) and elution (1M HNO_3) stage. As can be seen, peaks are not shaped or ranged as they are in best practice separations (**Figures 3, S4**).

Conclusions

Running the column dry with previously used, dry-stored columns is a combination that dramatically changes chromatographic performance of LN2 columns. The resulting elution patterns are irreproducible and make proper cutoff between rinsing to waste and collecting for a large separation factor nearly impossible. Columns should not be dry-stored and reused from previous separations. It is possible that wet-storage or repacking of the column could allow for the reuse of LN2 resin material.

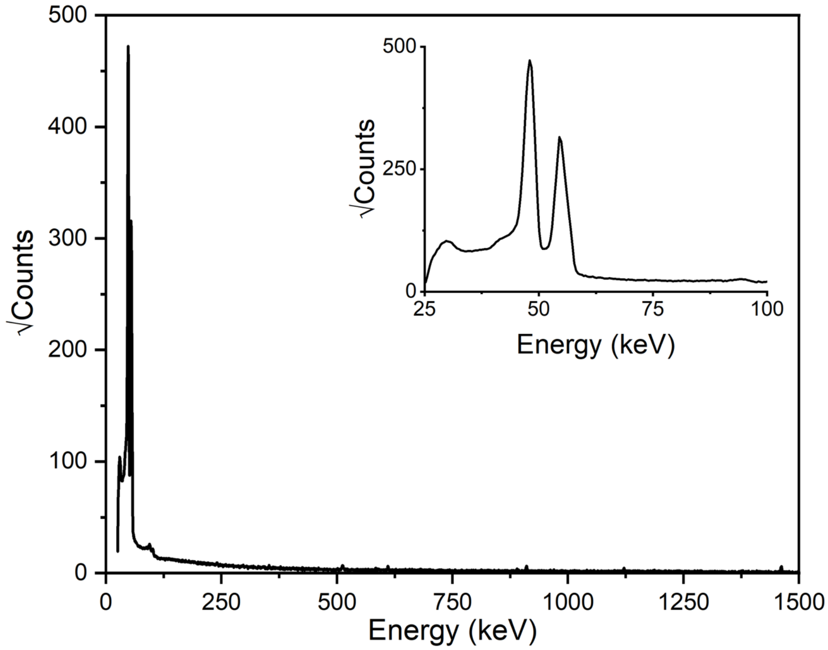

Supplementary Figure S6. High purity germanium gamma spectrum of representative purified ^{165}Er plotting the square root of the number of counts as a function of photon energy (keV).

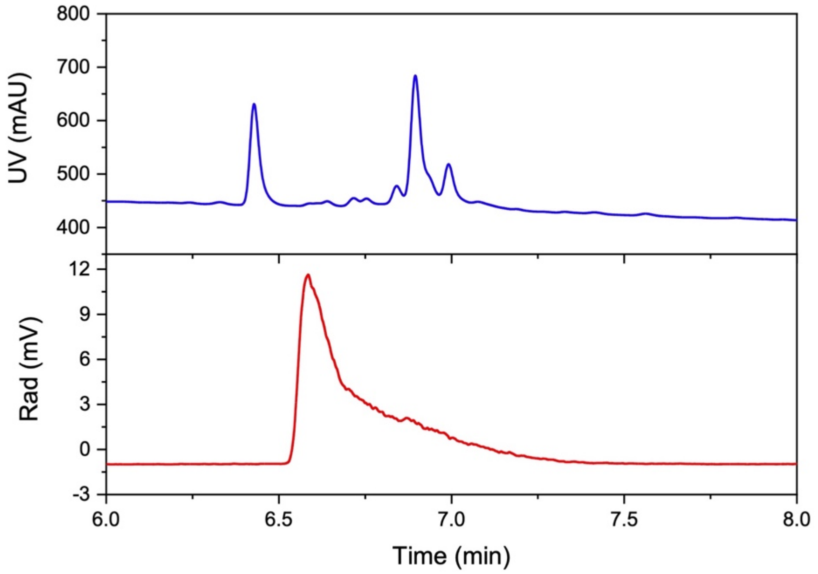

Supplementary Figure S7. Analytical HPLC radiochromatograph (top: 230 nm absorbance, bottom: radiation detector) of representative purified [^{165}Er]PSMA-617. *Mixing effects resulting from the transition from capillary (0.17 mm inner diameter) to wider bore tubing (0.8 mm inner diameter) at the UV detector caused the observed tailing in the downstream radiation detector signal.*"

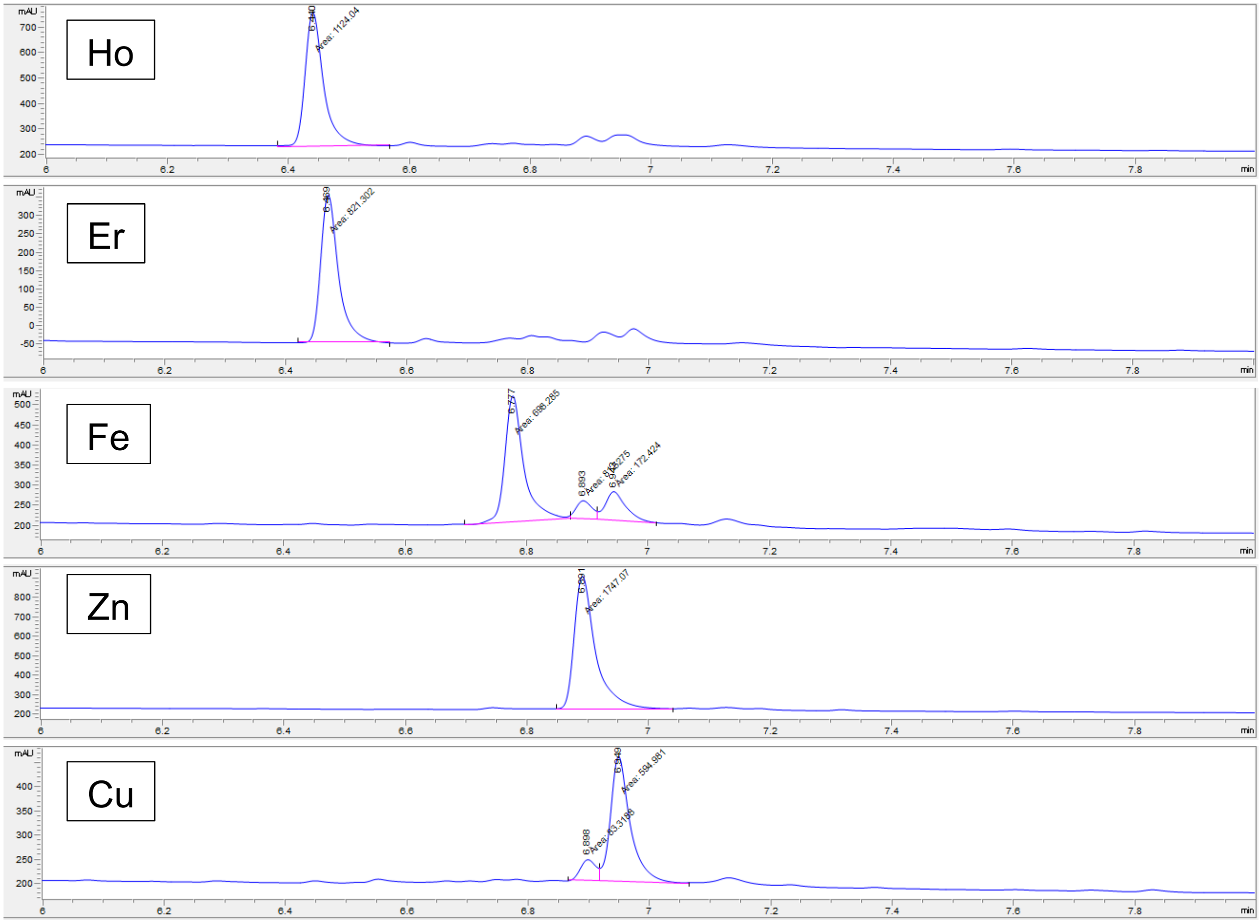

Supplementary Figure S8. Analytical HPLC chromatograph (230 nm absorbance) of cold metal PSMA-617 compounds. Each reaction contained NaOAc (50 µL, 1 M, pH 5.7), L-ascorbic acid (14 µL, 20 mg/mL), PSMA-617 (1 nmol, 10 µL, 0.1 µg/µL) and metal ion – Ho, Er (3 nmol, 50 µL in 2.4 mM HCl), Fe, Zn, or Cu (30 nmol, 50 µL in 6 mM HCl) and was heated for 30 minutes at 80 °C prior to analytical HPLC injection.

Supplementary Equation S1

Considering ^{165}Er mass, cold erbium target impurity, and residual holmium mass, the maximum achievable EOB molar activity (MA_{EoB} in MBq ^{165}Er per nmole of Ho/Er) of a final formulation of ^{165}Er can be calculated from the following parameters: EOB ^{165}Er radioactivity in MBq (A_{EOB}), Ho target mass in ng (m_{Ho}), fractional erbium impurity in Ho target (F_{ErImp}), fractional radiochemical yield of ^{165}Er (Y_{165Er}), Ho/Er separation factor ($SF_{Ho/Er}$), ^{165}Er decay constant in s^{-1} (λ), molecular weight of Ho in ng/nmol (MW_{Ho}), and Avogadro's number in atoms/nmol (N_{Av}) using equation (S1).

$$MA_{EoB} = \frac{A_{EoB} \cdot Y_{165Er}}{\left(\frac{A_{EoB} \cdot 10^6 \frac{Bq}{MBq} \cdot Y_{165Er}}{\lambda \cdot N_{Av}}\right) + \left(\frac{m_{Ho} \cdot F_{ErImp} \cdot Y_{165Er}}{MW_{Er}}\right) + \left(\frac{m_{Ho}}{MW_{Ho} \cdot \frac{SF_{Ho/Er}}{Y_{165Er}}}\right)} \quad \text{(S1)}$$

Supplemental Material References

[1] Tárkányi F, Hermanne A, Takács S, Ditrói F, Király B, Kovalev SF, et al. Experimental study of the $^{165}Ho(p,n)$ nuclear reaction for production of the therapeutic radioisotope ^{165}Er. Nucl Instruments Methods Phys Res Sect B Beam Interact with Mater Atoms 2008;266:3346–52. https://doi.org/10.1016/j.nimb.2008.05.005.

[2] Gracheva N, Carzaniga TS, Schibli R, Braccini S, van der Meulen NP. ^{165}Er: A new candidate for Auger electron therapy and its possible cyclotron production from natural holmium targets. Appl Radiat Isot 2020;159:109079. https://doi.org/10.1016/j.apradiso.2020.109079.

[3] Beyer GJ, Zeisler SK, Becker DW. The Auger-electron emitter ^{165}Er: excitation function of the $^{165}Ho(p,n)^{165}Er$ process. Radiochim Acta 2004;92:219–22. https://doi.org/doi:10.1524/ract.92.4.219.35608.
